# TLR4-Dependent DUOX2 Activation Triggered Oxidative Stress and Promoted HMGB1 Release in Dry Eye

**DOI:** 10.3389/fmed.2021.781616

**Published:** 2022-01-13

**Authors:** Bowen Wang, Hao Zeng, Xin Zuo, Xue Yang, Xiaoran Wang, Dalian He, Jin Yuan

**Affiliations:** State Key Laboratory of Ophthalmology, Zhongshan Ophthalmic Center, Guangdong Provincial Key Laboratory of Ophthalmology and Visual Science, Sun Yat-sen University, Guangzhou, China

**Keywords:** dry eye, DUOX2, oxidative stress, ROS, HMGB1, inflammation

## Abstract

Dry eye disease (DED) is one of the most common ocular surface diseases worldwide. DED has been characterized by excessive accumulation of reactive oxygen species (ROS), following significant corneal epithelial cell death and ocular surface inflammation. However, the key regulatory factor remains unclear. In this study, we tended to explore whether DUOX2 contributed to DED development and the underlying mechanism. Human corneal epithelial (HCE) cells were treated with hyperosmolarity, C57BL/6 mice were injected of subcutaneous scopolamine to imitate DED. Expression of mRNA was investigated by RNA sequencing (RNA-seq) and quantitative real-time PCR (qPCR). Protein changes and distribution of DUOX2, high mobility group box 1 (HMGB1), Toll-like receptor 4 (TLR4), and 4-hydroxynonenal (4-HNE) were evaluated by western blot assays and immunofluorescence. Cell death was assessed by Cell Counting Kit-8 (CCK8), lactate dehydrogenase (LDH) release, and propidium iodide (PI) staining. Cellular ROS levels and mitochondrial membrane potential (MMP) were analyzed by flow cytometry. RNA-seq and western blot assay indicated a significant increase of DUOX2 dependent of TLR4 activation in DED both *in vitro* and *in vivo*. Immunofluorescence revealed significant translocation of HMGB1 within corneal epithelial cells under hyperosmolar stress. Interestingly, after ablated DUOX2 expression by siRNA, we found a remarkable decrease of ROS level and recovered MMP in HCE cells. Moreover, knockdown of DUOX2 greatly inhibited HMGB1 release, protected cell viability and abolished inflammatory activation. Taken together, our data here suggest that upregulation of DUOX2 plays a crucial role in ROS production, thereafter, induce HMGB1 release and cell death, which triggers ocular surface inflammation in DED.

## Introduction

Dry eye disease (DED) is a global ocular surface disorder. The prevalence of DED worldwide ranged from 5 to 50% respectively (1). Generally, patients with DED frequently suffer from eye discomfort, dryness, and pain, which could influence work efficiency and finally vision-related quality of life (2). Until now, although the specific pathophysiological mechanism of DED is not fully established, one of the recognized core mechanisms is chronic ocular surface inflammatory responses (3). Therefore, targeting early inflammation activation may provide new therapeutic possibilities for DED treatment. Recently, several research have elucidated that consecutive damage of human corneal epithelium cells could give rise to ocular surface inflammation (4, 5).

Corneal epithelium is the outermost layer of the eye so that it sustains massive oxidative stress from the external environment (6). One of the outstanding characteristics of ocular surface oxidative stress is the overproduction of reactive oxygen species (ROS), particularly within human corneal epithelial (HCE) cells (7, 8). In our previous study, we have clarified that ROS accumulation in HCE cells could induce impaired autophagy, which further lead to cell death and the activation of inflammation (9). Whereas how risk factors of DED trigger oxidative stress accumulation and influence downstream cell viability remain uncertain.

Dual oxidase 2 (DUOX2) is a member of the nicotinamide adenine dinucleotide phosphate (NADPH) oxidase family that regulates the production of intracellular ROS, and plays diverse biological functional roles (10). DUOX2 is remarkably expressed in multiple epithelial cell types and has been implicated to perform host defense functions in various diseases (11, 12). DUOX2-mediated host defense is mainly derived from generating hydrogen peroxide and other oxides as well as inducing redox-dependent regulation of cellular responses (13, 14). Nevertheless, moderate activation of response pathway is beneficial to restore homeostasis, but excessive activation could conversely lead to the development of disease (15). It is remarkable, however, that the pathological effect of DUOX2 in ROS-cell death axis is still indistinct in DED.

High mobility group box 1 (HMGB1) is a highly conservative nucleoprotein (16). Nuclear HMGB1 serves in the regulation of DNA damage repair as a DNA chaperone. Interestingly, after post-translational modifications, HMGB1 could translocate from the nucleus to the cytoplasm, and thereby involved in cellular immune reaction (17, 18). Several research indicated that the elevation of HMGB1 within cytoplasm plays a key role in cell necrosis and production of pro-inflammation cytokine accelerating enormous inflammatory responses (19, 20). Though HMGB1 expression was found to be enhanced by dry eye conditions, the crosstalk between DUOX2-mediated excessive ROS release and HMGB1 during DED inflammation progression has not been previously investigated.

In the current study, we tended to explore the role of DUOX2 in DED and provide a novel therapeutic target for DED. As far as we are aware, our present research for the first time reveals that Toll-like receptor 4 (TLR4)-dependent increase of DUOX2 promotes oxidative stress in HCE cells, which triggers the cell death by means of enhancing translocation of HMGB1 and accelerates ocular surface inflammation in DED.

## Methods

### Reagents

Dulbecco's modified Eagle's medium (DMEM)/F12 medium, fetal bovine serum (FBS), penicillin/streptomycin, 0.25% trypsin-EDTA, recombinant human epidermal growth factor (hEGF), and an insulin-transferrin-selenium supplement were obtained from Invitrogen/Gibco (Carlsbad, CA, USA). Additionally, 6-, 12-, 24-, and 96-well culture plates and cell culture dish were purchased from Corning (Corning, NY, USA). Cell Counting Kit-8 (CCK8) was gained from Dojindo Laboratories (Japan). A Pierce LDH Cytotoxicity Assay Kit was purchased from Thermo Fishier Scientific (Rockford, IL, USA). DCFDA/H2DCFDA-Cellular ROS Assay Kit (#ab113851) and the anti-HMGB1 (#ab18256) antibody were purchased from Abcam (Cambridge, UK). The anti-4-HNE (#MA5-27570) was purchased from Invitrogen. The anti-DUOX2 (#sc-398681) antibody was obtained from Santa Cruz Biotechnology (TX, USA). The antibody targeting TLR4 (#A11226) was purchased from ABclonal Biotech (Wuhan, China). The following antibodies were purchased from Cell Signaling Technology (Danvers, MA, USA): anti-β-actin (#3700), anti-GAPDH (#5174), horseradish peroxidase (HRP)-conjugated anti-rabbit IgG (#7076), HRP-conjugated anti-mouse IgG (#7072), Alexa Fluor 488-labeled donkey anti-rabbit IgG (#4412), Alexa Fluor 488-labeled donkey anti-mouse IgG (#4409), and DAPI (#4083). Scopolamine hydrobromide (#427039), Lipopolysaccharides (LPS, #8643), and other reagent-grade chemicals were purchased from Sigma-Aldrich (St. Louis, MO, USA) unless otherwise indicated.

### Cell Culture and Development of Hyperosmolar Stress Model

Human SV40 immortalized corneal epithelial cell line [CRL-11135, human corneal epithelium (HCE2)] was purchased from ATCC (Manassas, VA, USA). HCE cells were cultured on culture dish in a humidified atmosphere of 5% carbon dioxide (CO_2_) at 37°C. Then, 5 μg/ml insulin, 10 ng/ml human epidermal growth factor, 10% fetal bovine serum, 1% penicillin/streptomycin, and DMEM/F12 composed the culture medium. After adherence, HCE-2 cells were treated for iso- or hyperosmolar (312 or 500 mOsM, respectively) culture medium. The hyperosmolarity was implemented by adding 94 mM sodium chloride.

### Cell Viability Assay

According to the protocol of manufacturer, CCK8 assay was used to detect cell viability. In brief, HCE cells were seeded in 96-well plates and exposed to a conditioned medium. After treatment, each well of the plates was added in 100 μl of a mixture of culture medium and CCK8 solution. The plate was incubated for 1–2 h in incubator (at 37°C and 5% CO_2_) afterward. A microplate reader (BioTek Instruments, Winooski, VT, USA) was used to measure the absorbance at 450 nm.

### Lactate Dehydrogenase Assay

Pierce^TM^ LDH Cytotoxicity Assay Kit (Thermo Scientific, #C20300) was applied to the measurement of lactate dehydrogenase (LDH) release. Briefly, HCE cells were cultured under a conditioned medium and equivoluminal of cell-free supernatant was added into a 96-well plate in triplicate. Then, LDH substrate mixture was added to each well and still standing at room temperature for 30 min. A microplate reader was used to evaluate the absorbance at 490 and 680 nm.

LDH release $=\;\frac{\;A_{490nm}\;of\;treated\;group-A_{680nm}\;of\;treated\;group}{A_{490nm}\;of\;control\;group-A_{680nm}\;of\;control\;group}$,where A is the absorbance.

### ROS Measurement

The level of ROS was measured using a 2',7'-dichlorofluorescein diacetate (H2DCFDA) assay kit as described in our previous study (21). In brief, HCE cells were seeded in 6-well plates. After adherence, the culture medium was replaced by a conditioned medium. When confluent, the cells were rinsed and incubated with 10 μM of H2DCFDA at 37°C for 30–45 min. Subsequently, the reaction mixture was replaced by PBS. The fluorescence intensity was evaluated by flow cytometry (BD LSRFortessa™ cell analyzer, NJ, USA).

### Mitochondrial Membrane Potential Assay

A TMRE-Mitochondrial Membrane Potential (MMP) Assay Kit (ab113852; Abcam) was used for MMP assay as described before (22). Briefly, after culture, cells were rinsed with PBS for 3 times and incubated with TMRE at 37°C for 15–30 min. Subsequently, the cells were resuspended with PBS and detected by flow cytometry.

### RNA Interference

The HCECs were transfected using Lipofectamine^TM^ RNAiMAX Transfection Reagent (Invitrogen, #13778150) with following siRNAs: siDUOX2, #1: sense, GGACAACAUAGUGGUUGAATT, antisense, UUCAACCACUAUGUUGUCCTT; #2: sense, CAGUCAAUGUCUACAUCUUTT, antisense, AAGAUGUAGACAUUGACUGTT; #3: sense, CAAAUGCUGUGUAAGAAGATT; antisense, UCUUCUUACACAGCAUUUGTT; si-TLR4, #1: sense: GCAAUUUGACCAUUGAAGATT, antisense, UCUUCAAUG GUCAAAUUGCTT; #2: sense, CAUUGGAUACGUUUCCUUATT, antisense, UAAGGAAACGUAUCCAAUGTT. #3: sense, GAAGUUGAACGAAUGGAAUTT, antisense, AUUCCAUUCGUUCAACUUCTT. Non-targeting scramble siRNA (si-NC) was applied as a negative control treatment. In brief, siRNA was dissolved in nuclease-free water to achieve a final concentration of 10 μM. Subsequently, siRNA and Lipofectamine RNAiMAX was added to Opti-MEM, respectively. Then, the solution of siRNA and Lipofectamine RNAiMAX was mixed. The mixture was maintained at room temperature for 5 min to form total complexed. After that, the mixture was added to the culture dish equally. The medium was replaced after 24 h.

### Western Blot Analysis

The cells total protein was extracted by the Minute Total Protein Extraction Kit (Invent Biotechnologies, MN, USA, #SD001). The Bicinchoninic Acid (BCA) Protein Assay Kit (Millipore, Billerica, MA, USA) was used to measure the total protein concentration. Then, equal amounts of protein samples were loaded onto sodium dodecyl sulfate-polyacrylamide gels and electrophoresed. The separated proteins in the gels were transferred to polyvinylidene fluoride membranes (Millipore, Bedford, MA, USA). After blocking the membranes with 5% non-fat milk in Tris-buffered saline with Tween-20 for 1–2 h at room temperature, the membranes were incubated with the appropriate primary antibodies overnight. After the membranes were rinsed thoroughly, they were further incubated with secondary antibodies for 1 h at room temperature. The blots were visualized using an enhanced chemiluminescence (ECL) kit according to the instructions of manufacturer. Western-blot gray scale results were used to semiquantitative analysis by using Image J software (NIH, MD, USA).

### Establishment of RNA Sequence Library and Data Processing

RNA extraction was performed according to the protocol of manufacturer by an RNeasy Plus Mini Kit (Qiagen, Valencia, CA, USA). The RNA sequence (RNA-seq) libraries were constructed with a TruSeq Stranded mRNA Library Prep kit (Illumina, CA, USA, #20020594) and sequenced on Illumina PE150 sequencers. Sequencing reads were mapped to hg19 using Star. Transcripts Per Million (TPM) values were called using RNA-Seq by Expectation-Maximization (RSEM). Differential gene expression was determined using DESeq2 and defined at log_2_-fold change >1 as well as *p* < 0.01.

### Quantitative Real-Time PCR (QPCR)

Total RNA from HCECs or intact corneas were extracted by an RNeasy Plus Mini Kit (Qiagen, Valencia, CA, USA) according to the instructions of manufacturer. Total RNA was quantified with a spectrophotometer (NanoDrop ND-1000; Thermo Fisher Scientific, Waltham, MA, USA). After synthesized with the PrimeScript RT Master Mix (TaKaRa, Japan), the cDNA was amplified with SYBR Green Supermix (Bio-Rad Laboratories, Inc., CA, USA) using the Light Cycler 480 Real-Time PCR System and designed primers. Glyceraldehyde-3-phosphate dehydrogenase (GAPDH) served as an internal reference gene. The following sequences of the primers were used: DUOX2: Forward, 5′-CTGGGTCCATCGGGCAATC-3′, Reverse, 5′- GTCGGCGTAATTGGCTGGTA-3′; TLR4: Forward, 5′-AGACCTGTCCCTGAACCCTAT-3′, Reverse, 5′-CGATGGACTTCTAAACCAGCCA-3′; HMGB1: Forward, 5′-TATGGCAAAAGCGGACAAGG-3′, Reverse, 5′-CTTCGCAACATCACCAATGGA-3′; IL-1β: Forward, 5′-CCTGGAGAGAGCTCGGACT-3′, Reverse, 5′-TGTGTGCCAACCTGATGCAG-3′; IL18: Forward, 5′-TCTTCATTGACCAAGGAAATCGG-3′, Reverse, 5′-TCCGGGGTGCATTATCTCTAC-3′; NLRP3: Forward, 5′-GATCTTCGCTGCGATCAACAG-3′, Reverse, 5′-CGTGCATTATCTGAACCCCAC-3′; CASPASE1: Forward, 5′-TTTCCGCAAGGTTCGATTTTCA-3′, Reverse, 5′-GGCATCTGCGCTCTACCATC-3′; ASC: Forward, 5′-TGGATGCTCTGTACGGGAAG-3′, Reverse, 5′-CCAGGCTGGTGTGAAACTGAA-3′; TNF-α: Forward, 5′-CCTGGAGAGAGCTCGGACT-3′, Reverse: 5′-TGTGTGCCAACCTGATGCAG-3′; NF-κB: Forward, 5′-AACAGAGAGGATTTCGTTTCCG-3′, Reverse, 5′-TTTGACCTGAGGGTAAGACTTCT-3′; GAPDH: Forward, 5′-CTCATGACCACAGTCCATGC-3′, Reverse, 5′-TTCAGCTCTGGGATGACCTT-3′.

### Immunofluorescence Staining

For cells immunofluorescence staining, HCECs were grown in 24-well-plates containing a glass sheet in each well. After different treatments, the cells were washed with PBS, fixed with 4% paraformaldehyde for 10 min, blocked with 3% BSA which contain 0.3% Triton X-100 for 1 h, and then, incubated overnight at 4°C with the primary antibodies. After washing, the cells were incubated for 1.5 h with secondary antibody. Images were observed with a confocal fluorescence microscope (LEICA DMi8, Germany).

For corneal immunofluorescence staining, mice were killed, and the eyeballs were excised. After fixed and embedded in paraffin, the eyeballs were sectioned. Slices were dewaxed by dimethylbenzene and then suffered high pressure for antigen retrieval. The remaining steps were performed as previously described in cells immunofluorescence staining.

### Animal Model and Treatment

A total of 20 female C57BL/6j mice aged 6–8 weeks were purchased from Beijing Vital River Laboratory Animal Technology Co. Ltd (Beijing, China). The mice of dry eye model were maintained in an environmentally controlled room that was maintained at ≤ 30% humidity and were exposed to a continuous air draft created by fans. The mice were given subcutaneous scopolamine hydrobromide (1.5 mg/0.3 ml; Sigma-Aldrich) injections three times per day for 5 consecutive days, as previously described (9). Control mice matched for age and sex were maintained at environment of 50–75% relative humidity. All animal experiments complied with the Association for Research in Vision and Ophthalmology Statement for the Use of Animals in Ophthalmic and Vision Research and were approved by the Ethics Committee of Zhongshan Ophthalmic Center, Sun Yat-Sen University (Guangzhou, China; approval ID: 2020-138).

### Sodium Fluorescein Staining of the Corneal Epithelium

Sodium fluorescein was administered to the inferior lateral conjunctival sac to evaluate the degree of corneal epithelial defects in the mice. The corneal epithelial images were acquired using a cobalt blue filter under a slit-lamp microscope image system (SL-D7/DC-3/MAGENet; Topcon, Tokyo, Japan) by two independent technicians. The quantification of corneal defects was carried out by Image J software. The covering area of corneal defects (%) = (fluorescein sodium positive area/the whole cornea) × 100%.

### Statistical Analysis

All data are expressed as the mean ± SD. Statistical analyses were performed using GraphPad Prism. Student's *t*-test was used to compare differences between groups. One- or two-way ANOVA followed by Bonferroni's *post-hoc* test was used for comparisons among three or more groups. The *p* < 0.05 was considered to indicate statistical significance.

## Result

### DUOX2 Expression Is Increased in DED

**Figure 1:**
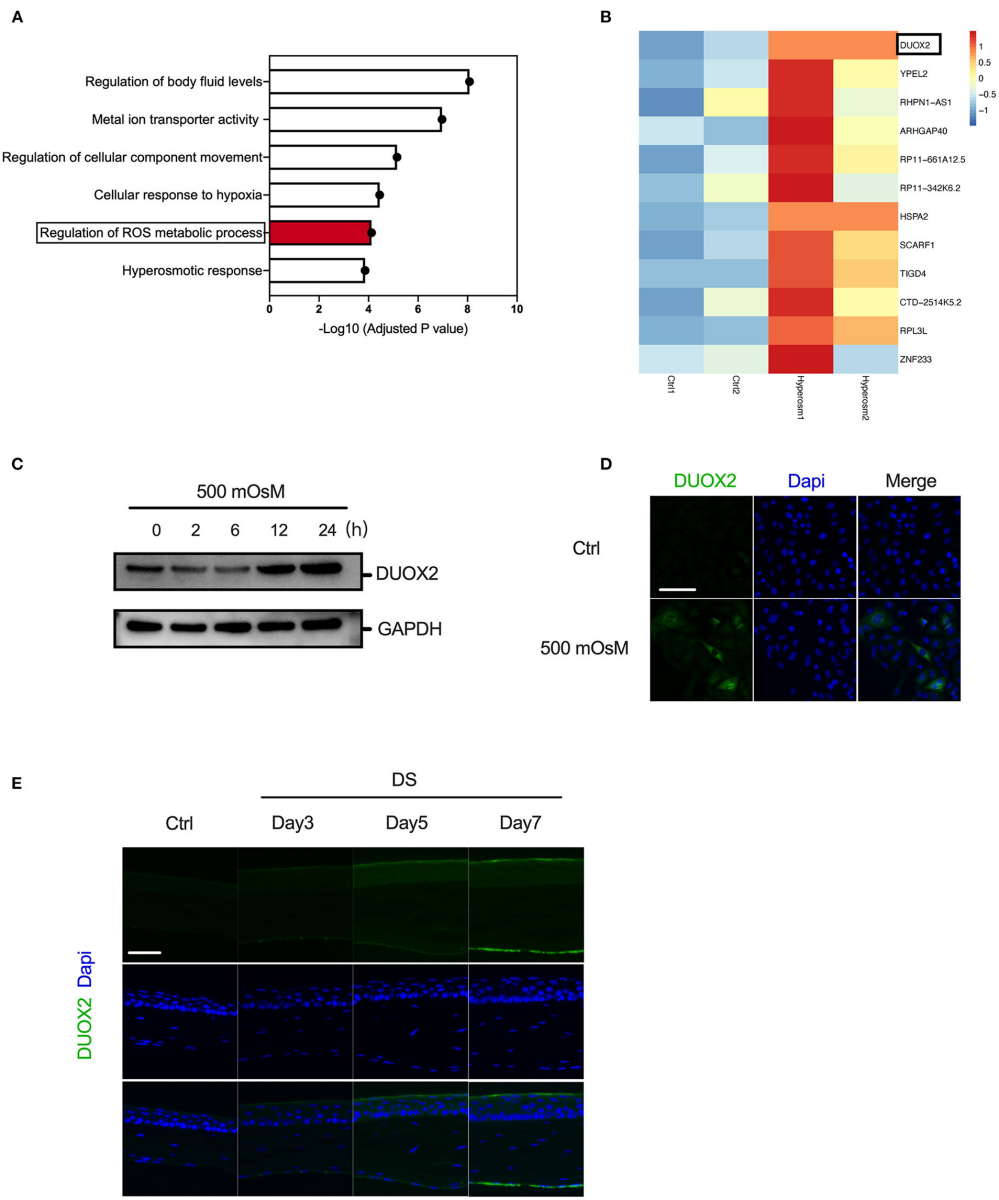
Activation of dual oxidase 2 (DUOX2) in dry eye disease (DED). **(A,B)** RNA sequencing (RNA-seq) from human corneal epithelial cells (HCECs) treated with/without hyperosmolar stress (500 mOsM) for 24 h showed differentially expressed genes related to oxidative stress. Biological process terms overexpressed for the oxidative-related genes identified in hyperosmolar stress. Top overexpressed Gene Ontology (GO) terms according to their fold-enrichment are depicted **(A)**. Heatmap showed the fold change of oxidative-related genes in control (Ctrl) and hyperosmolarity (Hyperosm) groups **(B)**. (Two samples from each group) **(C,D)** Western blot and immunofluorescence showing the changes of DUOX2 expression in human corneal epithelial (HCE) cells exposed to hyperosmotic medium (500 mOsM) relative to that in normal medium over time. **(E)** Immunofluorescence results showing the changes in DUOX2 expression in the corneal epithelium of desiccation stress (DS) mice relative to those in untreated (UT) mice after 3, 5, and 7 days and 5 mice were included in each group. Scale bar = 100 μm. The data shown are representative of two or more independent experiments.

Previous studies reported that excessive oxidative stress could lead to damage in HCE cell and contribute to DED development (23). Therefore, to further explore the key regulator of oxidative pathways in DED, we first carried out RNA-seq analysis in corneal epithelial cells exposed to hyperosmolarity. Our Gene Ontology (GO) analysis suggested that hyperosmolarity activated the regulation of body fluid levels, metal ion transporter activity, regulation of cellular component movement, and cellular response to hypoxia in HCE cells. Interestingly, we consistently found that there was a significant activated regulation of ROS metabolic process (Figure 1A). Moreover, in the detailed gene list, we discovered several oxidative-related regulators, such as DUOX2, YPEL2, and HSPA2, were greatly upregulated under hyperosmolarity comparing with the control group. Among them, the increase of DUOX2 was the most remarkably and stable during repeated experiment and then we chose DUOX2 for further investigation (Figure 1B). Moreover, we assessed the relative protein expression levels of DUOX2 using western blot, our results thereby confirmed an elevation of DUOX2 in HCE cells since 12 h after treatment of hyperosmolar stress (Figure 1C). In addition, immunofluorescence suggested a significant accumulation of DUOX2 after hyperosmolar treatment (Figure 1D). Then, to evaluate the change of DUOX2 *in vivo*, we used our established DED mouse model, which showed reduced tear aqueous secretion and significant decrease in the number of PAS-stained goblet cells in the conjunctiva (9). Consistently, we revealed DUOX2 was greatly increased within corneal epithelium in DED (Figure 1E). Taken together, our data here showed induction of DUOX2 in DED both *in vitro* and *in vivo*.

### TLR4 Is Essential for DUOX2 Activation Under Hyperosmolarity

**Figure 2:**
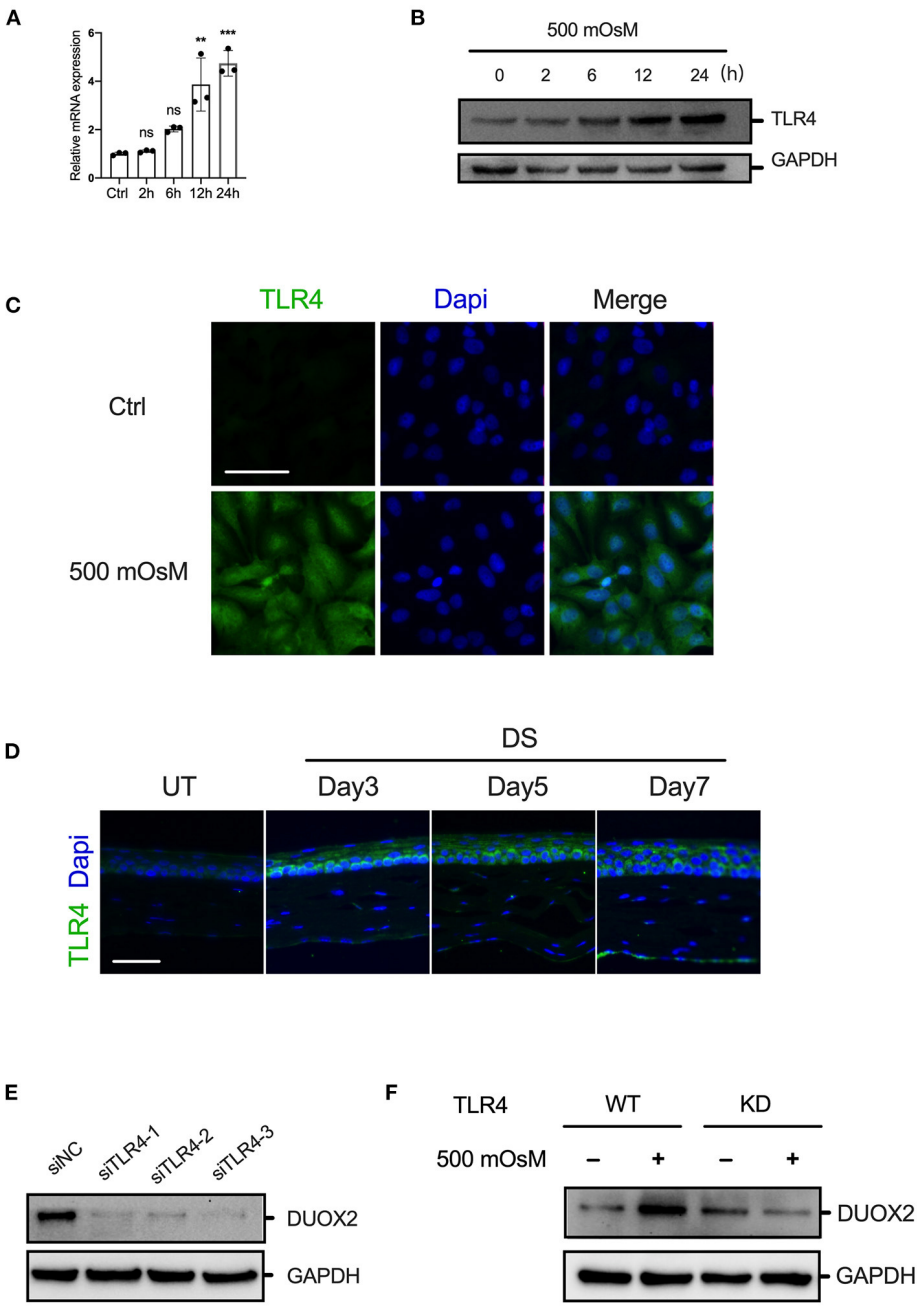
TLR4 is essential for DUOX2 activation under hyperosmolarity. **(A,B)** Human corneal epithelial cells were treated by hyperosmolarity (500 mOsM) for indicated time. The expression of TLR4 was determined by RT-PCR and western blotting. **(C)** Representative fluorescence images showing the increased expression of TLR4 in HCE cells exposed to 500 mOsM or normal medium (Ctrl) after 6 h. **(D)** Immunofluorescence results showing the changes of TLR4 in the corneal epithelium of DS mice relative to those in UT mice after 3, 5, and 7 days and 5 mice were included in each group. **(E)** HCE cells were transfected with different siRNAs targeted to TLR4 or negative control (siNC) for 24 h. The expression of DUOX2 was determined by western blot. **(F)** Wild-type (WT) and TLR4-knockdown (KD) HCECs were exposed to hyperosmotic medium (500 mOsM) or normal medium (Ctrl) for 24 h. Expression levels of DUOX2 were measured by western blot assay. Scale bar = 100 μm. The data shown are representative of two or more independent experiments (mean ± SD). **p < 0.01 and ***p < 0.001 vs. untreated controls.

As several published papers have reported that induction of DUOX2 could be triggered *via* TLR4, which is a main modulator in cellular homeostasis (13). In our current study, RT-PCR and western blot showed that the expression of TLR4 was increased in HCE cells under hyperosmolarity (Figures 2A,B). Furthermore, immunofluorescence suggested that TLR4 was significantly activated in DED cell and mouse model (Figures 2C,D). Therefore, to test the crosstalk of TLR4 upon DUOX2, we tried to ablate TLR4 expression in HCE cells. After application of RNA interference system, in which control siRNA or three distinct human-specific siRNAs targeting different domains of TLR4 were transiently transfected into HCE cells. After 24 h following transfection, RT-PCR displayed that TLR4 siRNA knockdown TLR4 expression with high efficiency (Supplementary Figure 1). Interestingly, our data discovered that ablation of TLR4 was sufficient to suppress the increase of DUOX2 in HCE cells exposed to normal culture medium as well as hyperosmolarity, whereas control siRNAs had no inhibitory effects (Figures 2E,F). In contrary, we applied inducer of TLR4, LPS (1 μg/ml) to treated HCE cell. RT-PCR showed LPS could promote ~3-fold over the levels of DUOX2 in HCE cells comparing with the control group (Supplementary Figure 2). Together, our results indicated that TLR4 is necessary for synergistic increase of DUOX2 in DED.

### Induction of DUOX2 Increases Oxidative Stress in HCE Cells

**Figure 3:**
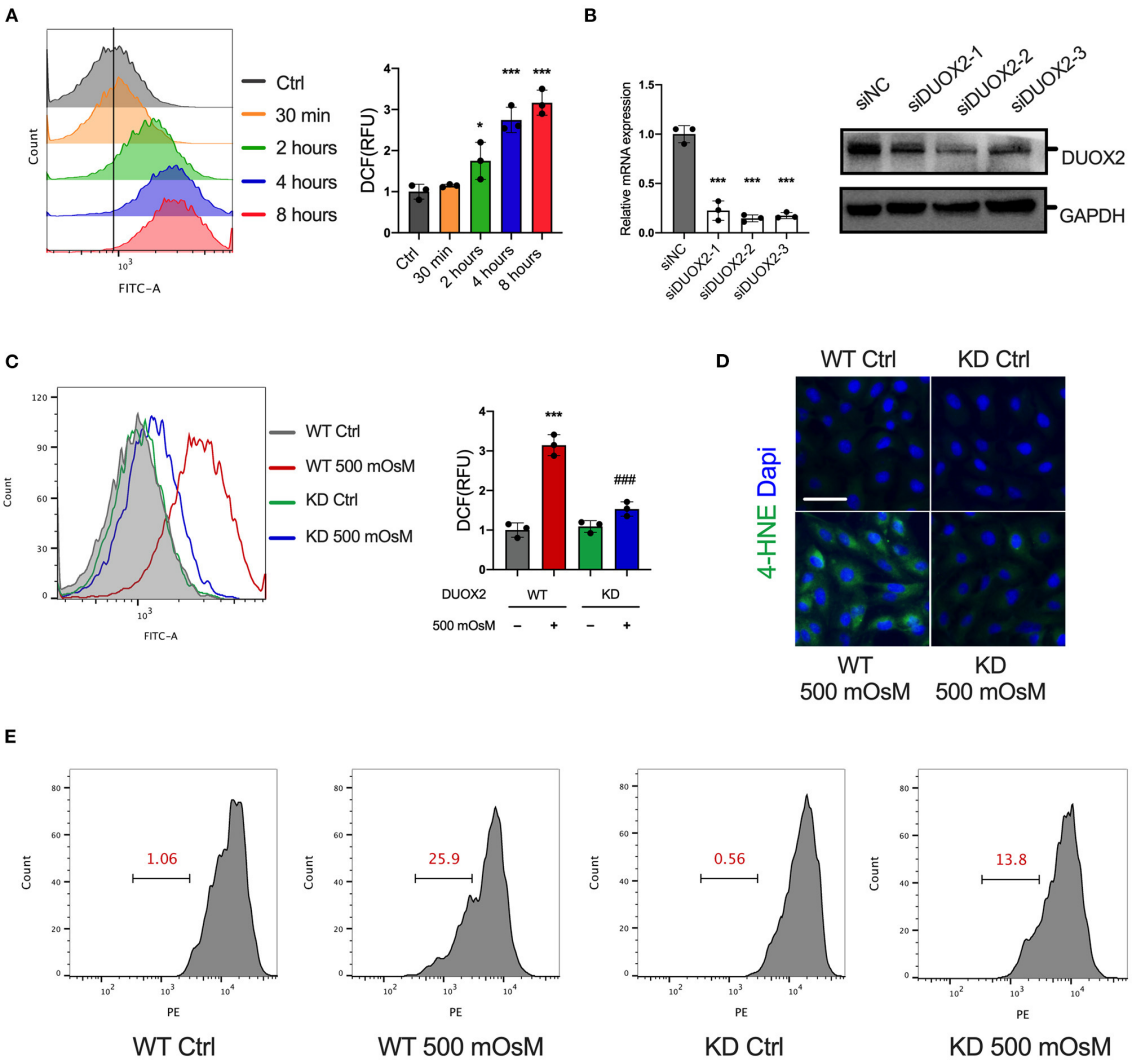
Elevation of DUOX2 triggers oxidative stress in HCE cells. **(A)** Reactive oxygen species (ROS) levels were measured in HCE cells exposed to normal medium (Ctrl) or hyperosmotic medium (500 mOsM) for the indicated times by 2′,7′-dichlorofluorescein diacetate (H2DCFDA) using flow cytometry. Right: relative fluorescence units (RFUs) were monitored. **(B)** HCE cells were transfected with different siRNAs targeted to DUOX2 or negative control (siNC) for 24 h. The expression of DUOX2 was examined by RT-PCR and western blot. Wild-type (WT) and DUOX2-knockdown (KD) HCE cells were exposed to hyperosmotic medium (500 mOsM) or normal medium (Ctrl) for 6 h. **(C)** ROS levels were measured by flow cytometry. Right: RFUs were monitored. **(D)** Representative fluorescence images showing the change of 4-hydroxynonenal (4-HNE). **(E)** Mitochondrial membrane potential (MMP) was evaluated by TMRE staining using flow cytometry. Scale bar = 100 μm. The data shown are representative of three or more independent experiments (mean ± SD). *p < 0.05 and ***p < 0.001 vs. untreated controls; ^###^*p* < 0.001 vs. 500 mOsM(+) DDIT4 KD(–).

**Figure 4:**
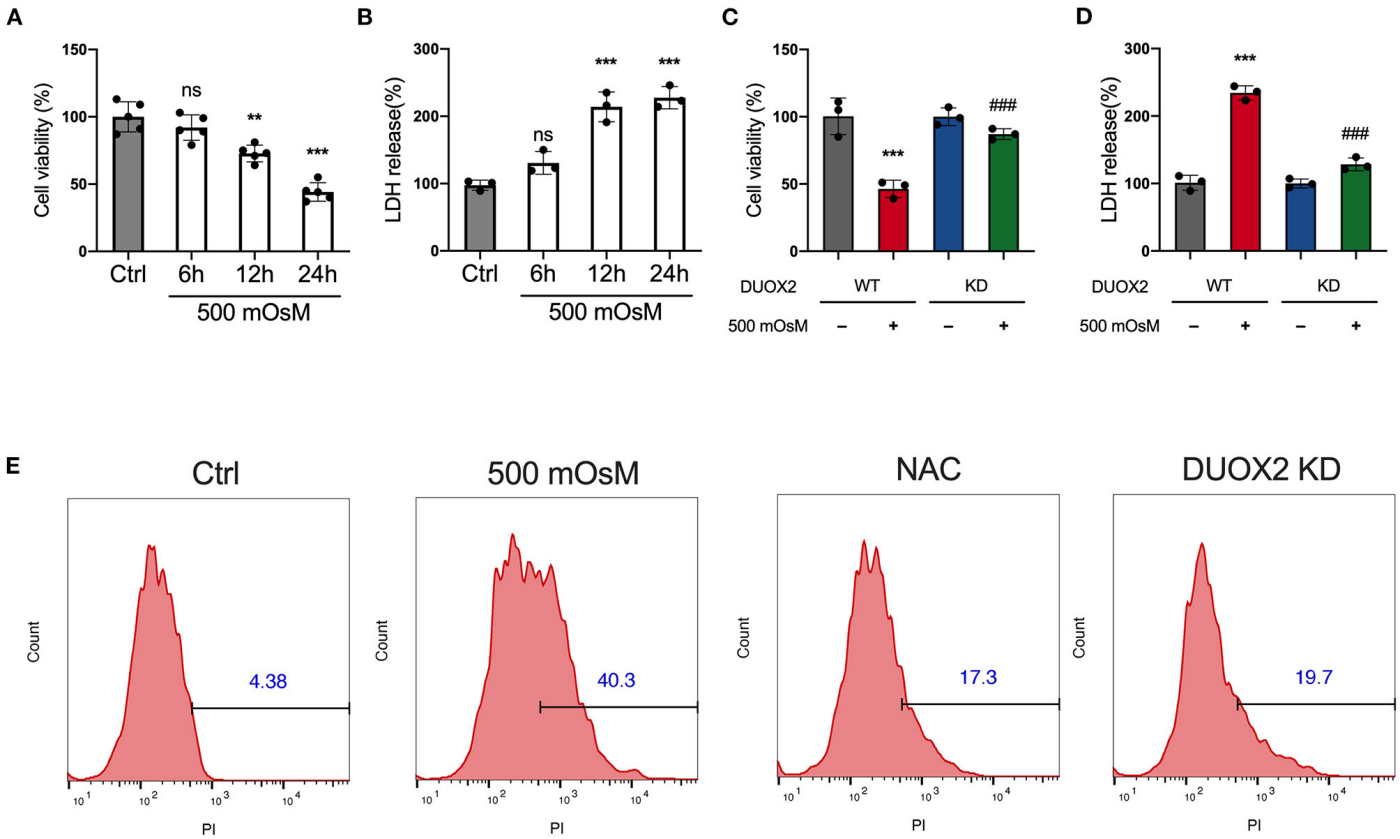
Ablation of DUOX2 inhibits hyperosmolarity-induced cell death. HCE cells were treated with 500 mOsM or normal culture medium (Ctrl) for 6, 12, and 24 h. **(A)** Changes in cell viability were determined by Cell Counting Kit-8 (CCK8) assay. **(B)** Changes in LDH levels were monitored by LDH assay. HCE cells were transfected with control siRNA (WT) or DUOX2 siRNA (KD) for 24 h and then treated with 500 mOsM or normal culture medium (ctrl) for 24 h. **(C)** Changes in cell viability were determined by CCK8 assay. **(D)** Changes in lactate dehydrogenase (LDH) levels were monitored by LDH assay. **(E)** HCE cells were transfected with DUOX2 siRNA (DUOX2 KD) for 24 h and cultured in hyperosmotic medium (500 mOsM) for 18 h. Control siRNA transfected HCE cells were exposed to 500 mOsM and co-treated with N-acetylcysteine (NAC) or left untreated (Ctrl). Positive percentage of propidium iodide (PI) staining was monitored by flow cytometry. The data shown are representative of three or more independent experiments (mean ± SD). **p < 0.01 and ***p < 0.001 vs. untreated controls; ^###^*p* < 0.001 vs. 500 mOsM(+) DUOX2 KD(–).

Considering the potential consequences of DUOX2 upon oxidative stress, we examined the time course of cellular ROS accumulation in DED. In response to hyperosmolar culture medium, the ROS production of HCE cells steadily increased over time (Figure 3A). We next measured that when DUOX2 was knockdown in HCE cells, the ROS level was changed to a much lesser degree under hyperosmolar challenge (Figures 3B,C). In addition, we observed remarkable mitochondrial dysfunction under hyperosmolar condition, which was detected *via* decreased MMP level. Surprisingly, these decreases were profoundly reversed by DUOX2 ablation (Figure 3E). Additionally, 4-hydroxynonenal (4-HNE) was considered as a potential mechanism by which ROS can inflict cellular damage within peroxidation cycle. We next observed a great reduction of 4-HNE expression in DUOX2-KD HCE cells exposed to hyperosmolarity comparing with wild type group (Figure 3D). To further investigate whether the increase of DUOX2 participated in cell fate decision in DED, we used CCK8 and LDH assay (Figures 4A,B). Our results turned out to find that inhibition of DUOX2 could significantly protect cell viability (Figures 4C,D). Moreover, this protective effect was consistently confirmed by declining PI positive cells in DUOX2-KD as well as an established ROS inhibitor N-acetylcysteine (NAC) treated HCE cells, respectively (Figure 4E). Therefore, our experiments implied here that induction of DUOX2 in DED had potential damage toward HCE cells through excessive oxidative stress.

### DUOX2 Promotes Translocation of HMGB1 and Aggregates Inflammation

**Figure 5:**
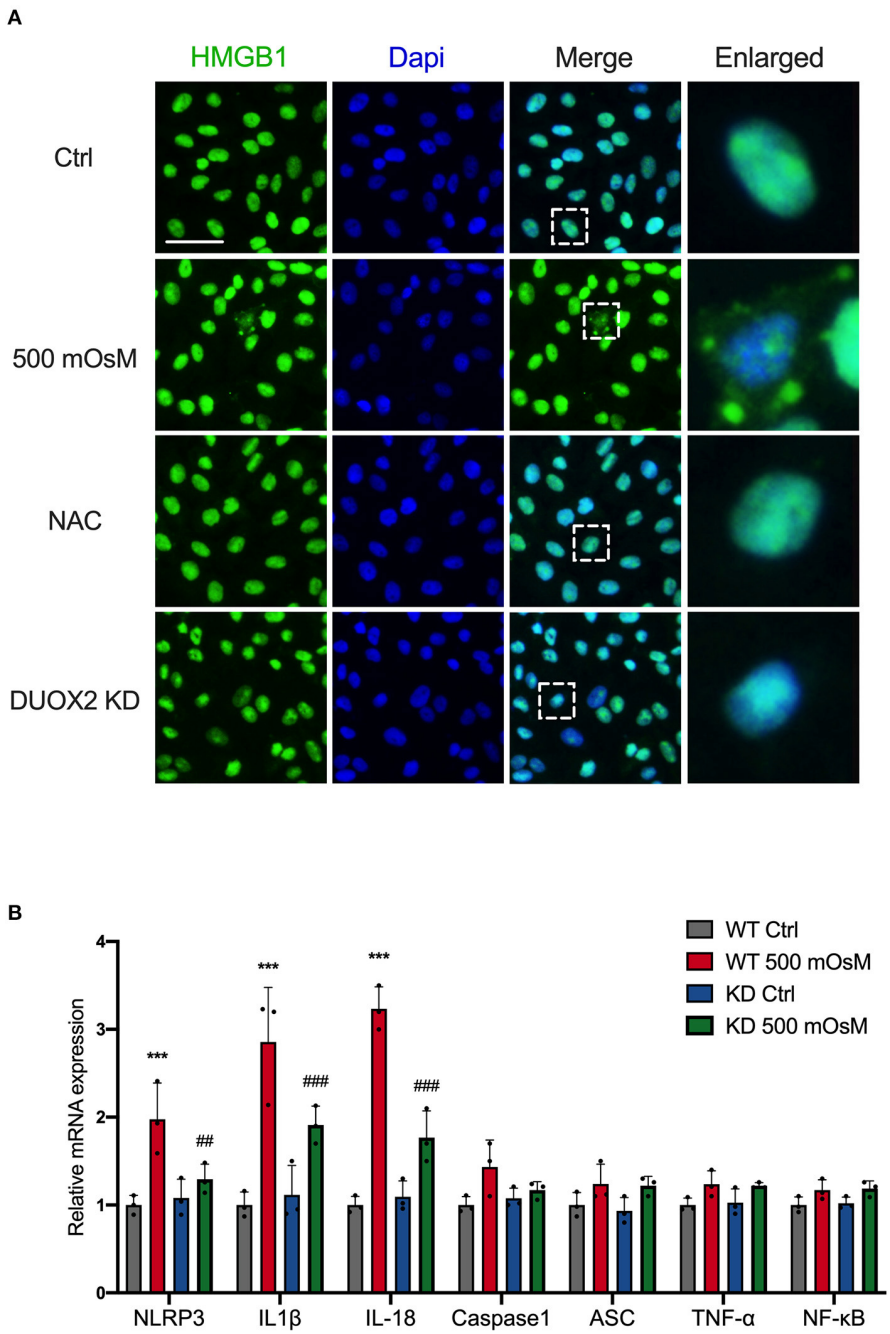
DUOX2-related oxidative pathway promotes translocation of high mobility group box 1 (HMGB1) and aggregates inflammation. **(A)** HCE cells were transfected with DUOX2 siRNA (DUOX2 KD) for 24 hours and cultured in hyperosmotic medium (500 mOsM) for 18 hours. Control siRNA transfected HCE cells were exposed to 500 mOsM and co-treated with NAC or left untreated (Ctrl). Representative fluorescence images showing the translocation of HMGB1. **(B)** HCE cells were transfected with control siRNA (WT) or DUOX2 siRNA (KD) for 24 hours and then treated with 500 mOsM or normal culture medium (Ctrl) for 24 hours. The mRNA changes of representative inflammation pathway were detected by RT-PCR. Scale bar = 100 μm. The data shown are representative of three or more independent experiments (mean ± SD). ***p < 0.001 vs. untreated controls; ^##^*p* < 0.01, ^###^*p* < 0.001 vs. 500 mOsM(+) DUOX2 KD(–).

Multiple cellular mechanisms known to regulate cell death through releasing and translocation of HMGB1 (24). To assess whether DUOX2 actively contributes to the change of HMGB1 in HCE cells, we first evaluate HMGB1 expression in DED. Surprisingly, RT-PCR found there was no obvious increase of HMGB1 in DED (Supplementary Figure 3). However, our immunostaining results showed that hyperosmolar treatment could cause obvious leakage of HMGB1 from the nucleus to the cytoplasm in HCE cells. By contrast, after knockdown of DUOX2, this translocation was effectively prevented. Considering that the elevation of oxidative stress could be responsible for HMGB1 translocation. We revealed ROS scavenger NAC was able to prohibit HMGB1 moving into cytoplasm (Figure 5A). Furthermore, HMGB1 is a chromatin-binding protein that could function as pro-inflammatory cytokines when released into extracellular space, we then explored the impacts of DUOX2-HMGB1 axis on inflammation pathways. Notably, we discovered that the mRNA expression levels of NOD-, LRR- and pyrin domain-containing 3 (NLRP3), interleukin-1β (IL-1β interleukin-18 (IL-18) in HCE cells were significantly attenuated after DUOX2 inhibition (Figure 5B). Collectively, we indicated that DUOX2 has potent regulatory effects upon cell death *via* enhancing translocation of HMGB1 and promotes ocular surface inflammation in DED.

## Discussion

Although DED is still one of the most common chronic ocular surface diseases, the specific underlying mechanism of pathogenesis remains to be clearly elucidated. Recently, a variety of studies have suggested that ocular surface oxidative stress is involved in the DED development (25, 26). In the present study, we discover that TLR4-mediated DUOX2 increase as a key regulator among oxidant pathways in DED. Furthermore, DUOX2 promotes cell death and inflammation activation through the enhancing of HMGB1 translocation.

In previous study, we demonstrated that inhibition of ROS could alleviate cell death in HCE cells (9). Using RNA-seq, we further screened and found that DUOX2 was highly expressed among oxidant pathways in HCE cells under hyperosmolarity. Moreover, this was further supported by immunofluorescence in our DED mouse model. However, the specific trigger DUOX2 in DED has not been investigated. It has been observed that corneal epithelial cells expressing TLRs are capable of signal transduction through TLR-related pathways, which previously recognized to be an exclusive characteristic of immune cells (27, 28). Interestingly, researchers recently revealed that ablated TLR4 signaling in HCE cells significantly decreased the development of ROS production and cell death (29, 30). Therefore, these results encourage our evaluation of TLR4 activation on the formation of DUOX2 in DED. As expected, the requirement for upregulation of TLR4-related signaling in the control of DUOX2 expression by hyperosmolarity was substantiated by the RNA interference studies. We noticed that siRNAs targeting different sites on TLR4 not only decreased TLR4 expression but also greatly inhibited DUOX2 expression at the RNA level, as well as the expression of DUOX2 protein. On the other hand, a well-established TLR4 inducer, LPS was able to prompt DUOX2 expression. We then confirm that induction of DUOX2 requires TLR4-mediated signaling, which provides new clues for DUOX2 regulatory network in ocular surface diseases. It is worth noting that GO analysis indicated fluid regulation and metal transportation were upregulated in HCE cells under hyperosmolarity. We assumed that future studies are needed to clarify the pathological connection between DUOX2 oxidative signaling and these biological procedures in dry eye development.

Interestingly, DUOX2 has been reported to play a critical role in host defense system, especially during chronic inflammatory conditions (10). In addition, it is reported that increase of DUOX2 could directly mediate the production of ROS, therefore accelerate development of gastrointestinal and respiratory diseases and cancer (31, 32). Our latest study discovered that a proper level of ROS was necessary for cell homeostasis maintenance, which could become important therapeutic strategy in DED treatment (33). In this research, we consistently discovered DUOX2 overexpression accompanied by excessive ROS production. Moreover, after inhibition of DUOX2, ROS release could be greatly abolished. DUOX2 knockdown could rescue the decrease of MMP, which is necessary to maintain normal mitochondrial function. These findings strongly suggest that a DUOX2-ROS axis exists in DED. Considering that the uncontrolled ROS release has been frequently combined with impaired cell function and cell death (34). We then found that DUOX2 ablation was able to protect cell viability and inhibit PI-positive cells under hyperosmolarity. Admittedly, more animal studies of DUOX2 knockout are necessary to further clarify the specific effect of DUOX2 in DED. Nevertheless, this present study provides new insight into potential DUOX2-based agents for future DED precision treatment. Moreover, based on our current database of RNA-seq, our results suggested that other oxidative-related genes, such as YPEL2 and HSPA2 could become promising treatment targets in dry eye after systematic *in vitro* and *in vivo* studies.

Notably, previous *in vitro* DED studies have suggested the co-existence of apoptosis and necrosis in oxidant-induced HCE injury (35, 36). In this study, we performed LDH analysis and revealed LDH release was strongly increased after hyperosmolar treatment, which could be decreased sharply in DUOX2 knockdown group. Hence, we indicated that DUOX2 might participate in the necrosis process of HCE cells in DED. Several observations demonstrate that abnormal release HMGB1 is highly pathogenic in the context of necrosis (37, 38). Indeed, our results suggested that although there was no obvious increase of HMGB1 in mRNA level, hyperosmolar stimulus could lead to the translocation of HMGB1 from the nucleus to the cytoplasm in HCE cells. Importantly, ablation of DUOX2 could function to stop this trend. Furthermore, it has been shown that HMGB1 can exhibit proinflammatory role during ocular surface damage and dry eye-related inflammation *via* interacting with soluble PAMPs and binding to TLRs (39). Our data further evaluated that DUOX2 inhibition attenuated upregulation of NLRP3, IL-1β, and IL-18. By contrast, a latest study suggested that the release of HMGB1 did not mediate DED-related inflammation in the corneal epithelium by directly inducing inflammatory cytokine production (40). Based on our data, we suppose that the proinflammatory role of HMGB1 within the corneal epithelium could be dependent on other alarmins or inflammatory molecules derived from cell disruption. Chen et al. indicated that GSDMD-dependent pyroptosis, as one of the programmed cell necrosis, is pivotal for subsequent amplification of inflammatory cascades in DED (41). Further studies are needed to precisely determine the role of HMGB1 within interactions between specific mode of cell death and ocular surface inflammation.

Taken together, our work showed that corneal epithelial expression of DUOX2 was remarkably elevated in DED both *in vitro* and *in vivo*. Moreover, we implicated that TLR4 activation was necessary to enhance DUOX2 transcription. When DUOX2 was blocked in HCE cells, excessive ROS production and damaged mitochondrial function were greatly reversed. Lastly, our results suggested that the cytoprotective function of DUOX2 inhibition might rely on abolished HMGB1 translocation, which further prevented ocular surface inflammation. Consequently, this research for the first time provided the evidence that DUOX2 could be a key regulatory factor in DED and further elaborate the pathogenetic mechanisms. Admittedly, further work is required to identify safe and effective ways to target this novel pathway, which is a critical step in developing future DED therapies.

## Data Availability Statement

The datasets presented in this study can be found in online repositories. The names of the repository/repositories and accession number(s) can be found below: https://figshare.com/, 10.6084/m9.figshare.16663891 https://figshare.com/, 10.6084/m9.figshare.16663951.

## Ethics Statement

The animal study was reviewed and approved by Ethics Committee of Zhongshan Ophthalmic Center, Sun Yat-sen University.

## Author Contributions

BW and HZ performed the experiments, analyzed the data, and wrote the manuscript. XZ, XY, XW, and DH performed the experiments and interpreted the results. JY designed the study and reviewed and revised the paper. All authors contributed to the article and approved the submitted version.

## Funding

The funding organization had no role in the design or execution of this study. Financial and material support for this paper was provided by grants from the National Natural Science Foundation of China to JY (82171015), BW (82101083); Guangdong Basic and Applied Basic Research Foundation to BW (2020A1515110019).

## Conflict of Interest

The authors declare that the research was conducted in the absence of any commercial or financial relationships that could be construed as a potential conflict of interest.

## Publisher's Note

All claims expressed in this article are solely those of the authors and do not necessarily represent those of their affiliated organizations, or those of the publisher, the editors and the reviewers. Any product that may be evaluated in this article, or claim that may be made by its manufacturer, is not guaranteed or endorsed by the publisher.
