# Supplementary material for: TLR4-Dependent DUOX2 Activation Triggered Oxidative Stress and Promoted HMGB1 Release in Dry Eye
Source: Front Med (Lausanne). 2022 Jan 13;8:781616. doi: 10.3389/fmed.2021.781616 (PMC8793023; doi:10.3389/fmed.2021.781616)
Supplement: Supplementary file 1 [file Data_Sheet_1.PDF]

Supplementary Material

1 Supplementary Data

All supplementary files are deposited to FigShare for permanent storage and receive DOI: 10.6084/m9.figshare.16663891 and 10.6084/m9.figshare.16663951.

2 Supplementary Figures

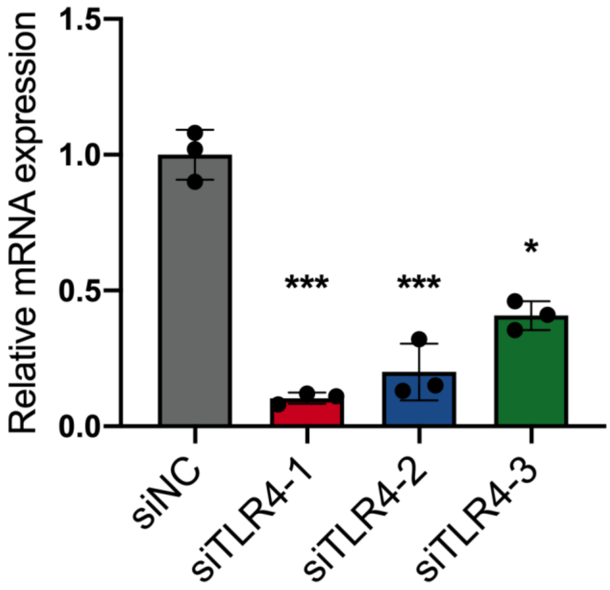

Supplementary Figure 1. Knockdown efficiency of TLR4-siRNA. HCE cells were transfected with three different TLR4 siRNA (DUOX2 KD) for 24 hours. Relative mRNA expression of TLR4 was determined by RT-PCR.

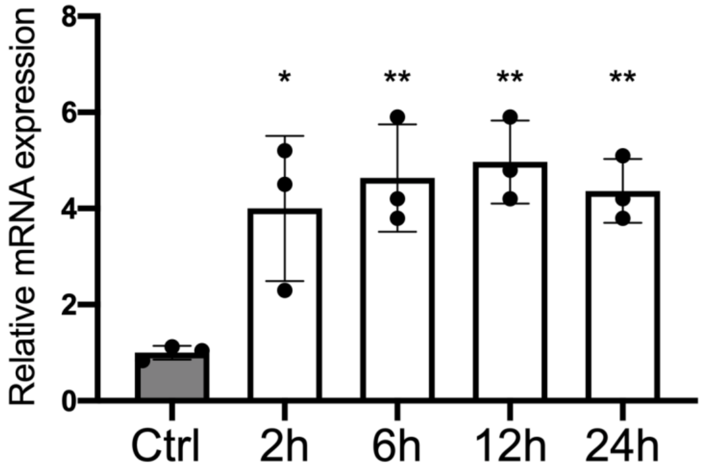

Supplementary Figure 2. LPS triggered expression of DUOX2 in HCE cells. HCE cells were treated with LPS for indicated times. Relative mRNA expression of DUOX2 was determined by RT-PCR.

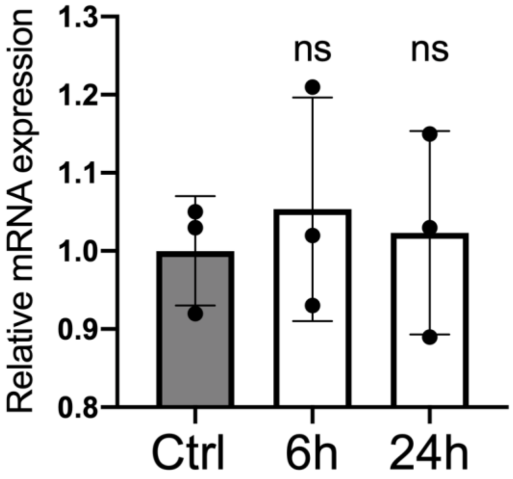

Supplementary Figure 3. HCE cells were cultured in normal medium or hyperosmotic medium (500 mOsM) for 6 and 24 hours. Relative mRNA expression of HMGB1 was determined by RT-PCR.
